# Publisher's Note - Pagination Error

**DOI:** 10.3390/s90604271

**Published:** 2009-06-04

**Authors:** Shu-Kun Lin

**Affiliations:** Molecular Diversity Preservation International (MDPI), Kandererstrasse 25, CH-4057 Basel, Switzerland; E-mail: lin@mdpi.org

*Publisher's Note* added on 4 June 2009: There is a pagination error in *Sensors* **2009**, *9*(6) with page 4271 missing. Thus, page 4271 is taken as a blank page.

